# Identification and Characterization of Circular Intronic RNAs Derived from Insulin Gene

**DOI:** 10.3390/ijms21124302

**Published:** 2020-06-17

**Authors:** Debojyoti Das, Aniruddha Das, Mousumi Sahu, Smruti Sambhav Mishra, Shaheerah Khan, Pruthvi R. Bejugam, Pranita K. Rout, Arundhati Das, Shehnaz Bano, Gyan Prakash Mishra, Sunil K. Raghav, Anshuman Dixit, Amaresh C. Panda

**Affiliations:** 1 Institute of Life Sciences (ILS), Nalco Square, Bhubaneswar, Odisha 751023, India; debojyotidas.phy@gmail.com (D.D.); ani.geneinvo@gmail.com (A.D.); mousumi.nina.sahu1@gmail.com (M.S.); sambhubioinfo@gmail.com (S.S.M.); khshaheerah@gmail.com (S.K.); raj.pruthvi@gmail.com (P.R.B.); pranita.liza.09@gmail.com (P.K.R.); arundhati.das12345@gmail.com (A.D.); j12mishra@gmail.com (G.P.M.); raghuvanshi2010@yahoo.co.in (S.K.R.); anshumandixit@gmail.com (A.D.); 2 School of Biotechnology, KIIT University, Bhubaneswar, Odisha 751024, India; 3 National Center for Cell Sciences (NCCS), Pune, Maharashtra 411007, India; sbs90786@gmail.com

**Keywords:** pancreatic β-cells, lariat-derived circRNA, insulin splicing, translation, diabetes

## Abstract

Circular RNAs (circRNAs) are a large family of noncoding RNAs that have emerged as novel regulators of gene expression. However, little is known about the function of circRNAs in pancreatic β-cells. Here, transcriptomic analysis of mice pancreatic islet RNA-sequencing data identified 77 differentially expressed circRNAs between mice fed with a normal diet and a high-fat diet. Surprisingly, multiple circRNAs were derived from the intron 2 of the preproinsulin 2 (*Ins2*) gene and are termed as circular intronic (*ci*)*-Ins2*. The expression of *ci-Ins2* transcripts in mouse pancreatic islets, and βTC6 cells were confirmed by reverse transcription PCR, DNA sequencing, and RNase R treatment experiments. The level of *ci-Ins2* was altered in βTC6 cells upon exposure to elevated levels of palmitate and glucose. Computational analysis predicted the interaction of several RNA-binding proteins with *ci-Ins2* and their flanking region, suggesting their role in the *ci-Ins2* function or biogenesis. Additionally, bioinformatics analysis predicted the association of several microRNAs with *ci-Ins2*. Gene ontology and pathway analysis of genes targeted by miRNAs associated with *ci-Ins2* suggested the regulation of several key biological processes. Together, our findings indicate that differential expression of circRNAs, especially *ci-Ins2* transcripts, may regulate β-cell function and may play a critical role in the development of diabetes.

## 1. Introduction

Diabetes is one of the most common diseases in the world [1]. Diabetes is characterized by increased blood glucose levels due to the dysfunctional sensing or production of insulin [2,3]. Pancreatic β-cells synthesize and secrete insulin to maintain glucose homeostasis in the body [4]. The biosynthesis and secretion of insulin from β-cells require tight regulation of gene expression at transcriptional as well as at post-transcriptional level [5,6,7]. It has been well established that transcription factors, RNA-binding proteins (RBPs), long noncoding (lnc)RNAs, and micro (mi)RNAs regulate insulin synthesis and secretion from β-cells [5,6,7,8,9]. In recent years, another class of ncRNA called circular RNA (circRNA) has emerged as a critical regulator of gene expression [10].

High-throughput RNA-sequencing (RNA-seq) has led to genome-wide annotation of circRNAs in a variety of organisms, including humans [11,12,13]. The majority of circRNAs are generated by the backsplicing of pre-mRNAs, whereby a downstream splice site covalently joins with an upstream splice site [13,14,15]. Additionally, some intronic lariats escape the debranching process during splicing and generate stable circular intronic (ci)RNAs [16]. CircRNAs are categorized into various types based on their sequence composition, such as exonic circRNA, exon-intron circRNA (EIciRNA), and circular intronic RNA (ciRNA) [13,16,17,18]. CircRNAs are resistant to exonucleases and are much more stable than the linear RNAs due to lack of free ends [19,20]. Recently, circRNAs have been reported to regulate gene expression by sponging miRNAs and RBPs, and by producing peptides [21,22,23,24]. Although the molecular mechanisms for the majority of the circRNAs are unknown, they have indeed been implicated as regulators of crucial cellular events, including signaling, proliferation, apoptosis, differentiation, and aging [23,25,26]. Furthermore, altered expression and function of circRNAs have been reported in various pathological conditions, including cancer, diabetes, Alzheimer’s disease, neurodegeneration, and muscle degeneration [27,28,29,30,31,32]. However, the function of circRNAs in β-cell physiology and the development of diabetes is not well studied.

In this study, we wanted to identify circRNAs expressed in pancreatic islets of a diabetes mice model and to investigate their role in β-cell physiology. Here, we used previously published RNA-seq data on pancreatic islets of a type 2 diabetes mouse model to identify the expression pattern of circRNAs. This comprehensive analysis of circRNAs with CIRCexplorer2 and CIRI2 identified thousands of circRNAs, and several circRNAs were found to alter their expression in the islets of mice fed with a high-fat diet (HFD) compared to a normal diet (ND). Interestingly, several circRNAs were found to be derived from the preproinsulin 2 (*Ins2*) gene. Furthermore, we validated the expression of *ci-Ins2* in mouse pancreatic islets and βTC6 cells. A computational analysis suggested that *ci-Ins2* transcripts might sponge several RNA-binding proteins (RBPs) and microRNAs. Together, our findings provide the first example of circular transcripts generated from the insulin gene and their potential to regulate β-cell physiology.

## 2. Results

### 2.1. Identification and Characterization of Pancreatic Islet CircRNAs

**Figure 1:**
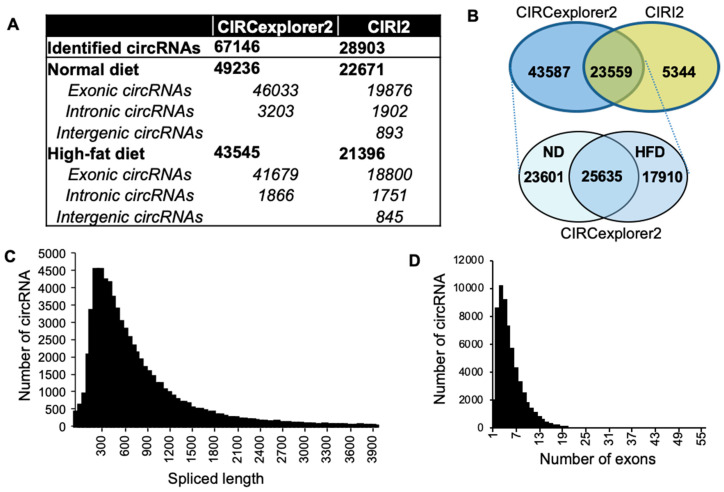
Annotation and characteristics of circular RNAs (circRNAs) in mouse pancreatic islets. (**A**) Summary of circRNAs identified in pancreatic islets of mice fed with normal diet (ND) and high-fat diet (HFD), using CIRCexplorer2 and CIRI2. (**B**) Venn diagram showing the overlapped circRNAs detected in pancreatic islets using CIRI2 and CIRCexplorer2 (*top*). Venn diagram of circRNAs detected by CIRCexplorer2 in pancreatic islets of mice fed with ND or HFD (*bottom*). (**C**) Distribution of the spliced length of circRNAs with length up to 4000 nucleotides in pancreatic islets detected by CIRCexplorer2. (**D**) The exon number distribution for exonic circRNAs detected with CIRCexplorer2.

To conduct the genome-wide identification of circRNAs in pancreatic islets, we retrieved publicly available RNA-seq data sets from pancreatic islets of mice fed with ND or HFD [33]. A total of 67,146 and 28,903 circRNAs were identified with circRNA annotation algorithms CIRCexplorer2 and CIRI2, respectively (Figure 1A,B). The vast majority of the circRNAs identified with CIRI2 and CIRCexplorer2 were derived from exonic sequences, while only about 10% of the circRNAs originated from intronic or intergenic regions. Since the majority of the circRNAs identified by CIRI2 were also identified in CIRCexplorer2 and the total number of identified circRNAs were significantly higher in CIRCexplorer2 annotation (Figure 1B, Tables S2 and S3), we used the CIRCexplorer2 data for further characterization of circRNAs.

Approximately half of the circRNAs identified with CIRCexplorer2 were exclusively expressed either in ND or in HFD samples (Figure 1B). The mature spliced length of identified circRNAs range from less than a hundred to several thousand nucleotide long, and the majority of the circRNAs were found to be less than 2000 nucleotides (Figure 1C). Although the number of exons incorporated in the exonic circRNAs varied from 1 to 56, the majority of them consisted of less than 15 exons (Figure 1D). Additionally, we determined the number of circRNAs derived from each chromosome (Figure S1A,B). The exonic circRNAs were found to prefer the second exon of the linear transcript as the starting exon; however, the end exon of circRNAs did not show any preference (Figure S1C).

Since pancreatic islets contain alpha, beta, delta, and PP cells, we used mouse pancreatic βTC6 cell line for validation of a selected list of circRNAs from RNA-seq analysis (Figure S2A). Additionally, we analyzed their abundance in βTC6 cells using RT-qPCR analysis (Figure S2B). Furthermore, the selected circRNAs were PCR-amplified from mouse pancreatic islets and βTC6 cells using specific divergent primers and were assessed on agarose gel followed by Sanger-sequencing to confirm the amplification of backsplice junction sequences (Figure S3A,B). Furthermore, an RNase R treatment assay was employed to check the circularity of the selected circRNAs. Indeed, RNase R treatment degraded the linear RNAs, while the tested circRNAs showed resistance to RNase R digestion (Figure S3C).

### 2.2. Identification of Differentially Expressed (DE) CircRNAs

**Figure 2:**
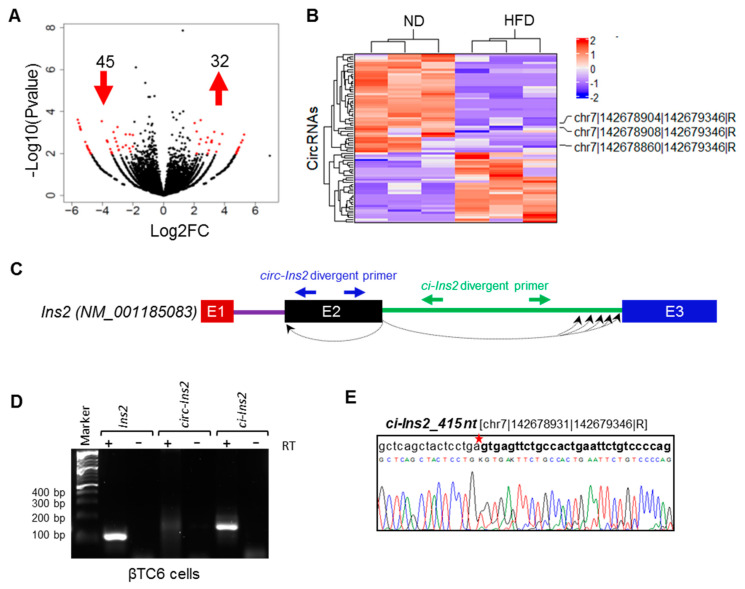
Differential expression and validation of circular RNA derived from the insulin 2 gene. (**A**) Volcano plot of differentially expressed circRNAs detected by CIRCexplorer2 in pancreatic islets of mice fed with HFD compared to ND. The log2 fold-change greater than 2 and *p*-value less than 0.01 were considered as differentially expressed. The upward and downward arrows indicate up- and downregulated circRNAs, respectively. (**B**) Heatmap representing hierarchical clustering of differentially expressed circRNAs, as mentioned in panel A. Three circRNAs derived from *Ins2* are labeled on the heatmap. (**C**) Schematic representation of mouse *Ins2* pre-mRNAs and the location of divergent primers for amplification of *circ-Ins2,* and *ci-Ins2.* The boxes represent the exons, and the lines represent the introns. The dotted curved lines represent the backsplicing or intron lariat junctions. (**D**) The RT-PCR products of *Ins2, circ-Ins2,* and *ci-Ins2* in βTC6 cells resolved and visualized in SYBR Gold-stained 2.5% agarose gel. (**E**) PCR products of *ci-Ins2* from βTC6 cells were purified and Sanger-sequenced to confirm the junction sequences of *ci-Ins2* transcripts.

To explore the molecular regulation of circRNAs in pancreatic islets, we sought to explore expression profiles of circRNAs in the pancreatic islets of mice fed with HFD compared with ND. Differential expression analysis identified 32 upregulated and 45 downregulated circRNAs in HFD islets compared to ND islets (Figure 2A). As shown in Figure 2B, the differentially expressed circRNAs were clustered based on their expression profiles in ND and HFD samples (Table 1). Interestingly, three of the circRNAs generated from mouse *Ins2* were downregulated in islets of mice fed with HFD compared to ND, suggesting a possible role for these circRNAs in the development of diabetes.

### 2.3. Multiple Exonic and Intronic CircRNAs are Generated from Ins2 Genes

Previous research has shown that alternative splicing and backsplicing events can generate multiple exonic and intronic circRNAs from a single gene [12,34]. Furthermore, circRNA biogenesis is involved in alternative splicing of the host gene, which could lead to altered gene expression [24]. Here, our RNA-seq data analysis using CIRCexplorer2 detected multiple exonic and circular intronic lariat RNAs (ciRNA) derived from insulin genes in mice (Table S4). Surprisingly, the RNA-seq data identified 39 circRNAs generated from the mouse *Ins2* gene, out of which only two are generated from exon2 while the rest is generated from intron2 of *Ins2* pre-mRNA. RT-PCR amplification of the circular exonic and intronic RNAs, with specific divergent primers in βTC6 cells, confirmed the expression of *circ-Ins2* and *ci-Ins2* transcripts (Figure 2C,D). PCR with RT and no-RT controls indicated that *circ-Ins2* and *ci-Ins2* are circular transcripts generated from *Ins2* pre-mRNA. However, the expression of *circ-Ins2* was very low and barely amplified in βTC6 cells and mice pancreatic islets (Figure S4). Furthermore, Sanger sequencing of the *circ-Ins2* PCR products could not consistently confirm any specific circular transcript from exon2 of *Ins2* (data not shown).

**Figure 3:**
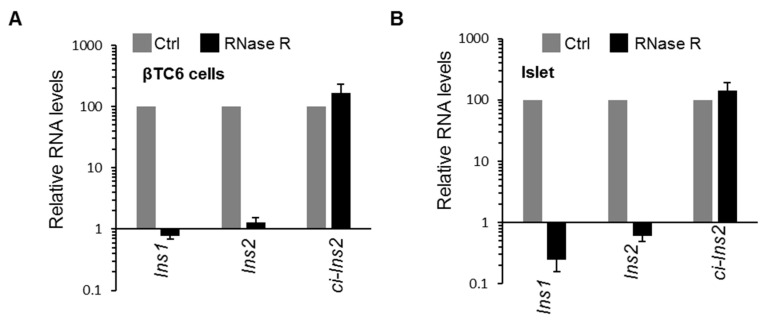
Circular nature and stability of *ci-Ins2*. (**A**,**B**) RT-qPCR analysis showing the levels of mRNAs and *ci-Ins2* upon RNase R treatment in βTC6 cells (**A**) and pancreatic islets (**B**). Data represent the means ± SEM from three independent experiments.

Although our RNA-seq data analysis detected 37 *ci-Ins2* RNAs, RT-PCR using divergent primer targeting the intron2 of *Ins2* gene amplified a single major product (Figure 2D). Sanger sequencing of the PCR products detected the *ci-Ins2* [chr7|142678931|142679346|R] with a length of 415 nt (Figure 2E). Since the RNA-seq predicted many *ci-Ins2* transcripts, the PCR products were cloned and sequenced to verify the *ci-Ins2* lariat junction sequence. Consistent with a previous publication, the lariat branch point showed a mismatch due to error incorporated during reverse transcription at the 2′-5′ lariat junction (Figure S5) [35]. Intronic lariat derived ci-RNAs are known to be very stable due to their resistance to debranching (DBR) enzymes as well as other exonuclease activity [16,36]. Here, we checked the circularity of *ci-Ins2* by digesting the total RNA from βTC6 cells and mouse pancreatic islets with RNase R exonuclease. Indeed, RNase R treatment degraded linear *Ins1* and *Ins2* mRNAs, not the *ci-Ins2* in both βTC6 cells and pancreatic islets (Figure 3A,B). These analyses suggest that *ci-Ins2* transcripts are bona fide stable circular transcripts.

### 2.4. Alternate Branchpoint Selection Generates Multiple Ci-Ins2 Lariats

**Figure 4:**
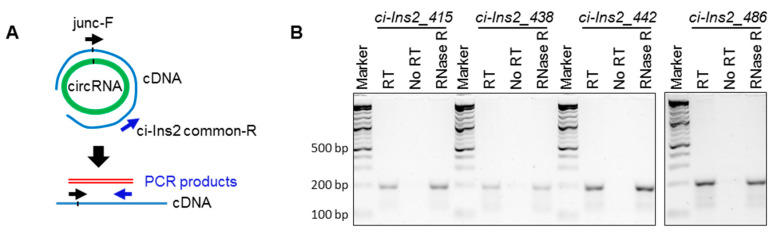
Multiple *ci-Ins2* transcripts are expressed in βTC6 cells. (**A**) Schematic representation of RT-PCR verification of circular RNAs, with the forward primer spanning the backsplice junction. Green circle represents the circRNA template, the blue line represents cDNA, arrows represent the PCR primers, and the red line represents expected PCR products. (**B**) PCR products of individual circular RNAs in βTC6 cells total RNA cDNA (RT), no-RT, and RNase-R-treated samples were resolved on 2.5% agarose gel stained with SYBR-Gold.

Multiple *ci-Ins2* transcripts were generated from Ins2 pre-mRNA, and three of them were differentially expressed in HFD (Table 1 and Table S4). Since divergent primers used against *ci-Ins2* could amplify multiple circular transcripts, we tried to perform RT-PCR analysis for the *ci-Ins2_415* [415 nt; chr7|142678931|142679346|R], *ci-Ins2_438* (438 nt; chr7|142678908|142679346|R], *ci-Ins2_442* [442 nt; chr7|142678904|142679346|R], and *ci-Ins2_486* [486 nt; chr7|142678860|142679346|R], using the primer spanning the backsplice junction sequence. As shown in Figure 4A, the forward primer spans the junction targeting specific *ci-Ins2* transcripts, while the reverse primer was common for all four transcripts tested here. RT-PCR analysis of these ci-Ins2 transcripts using RT, no-RT, and RNase-R-treated samples from βTC6 cells suggested the expression of multiple *ci-Ins2* transcripts in beta cells (Figure 4B). Interestingly, all the *ci-Ins2* variants start at 5′ splice site of intron 2 of *Ins2* pre-mRNA, while the end is variable (Figure S6). These variants may be generated due to alternative branchpoint selection during *Ins2* pre-mRNA splicing, consistent with previous findings [37].

### 2.5. Exposure of βTC6 Cells to Palmitate and High Glucose Alters the Level of Ci-Ins2

**Figure 5:**
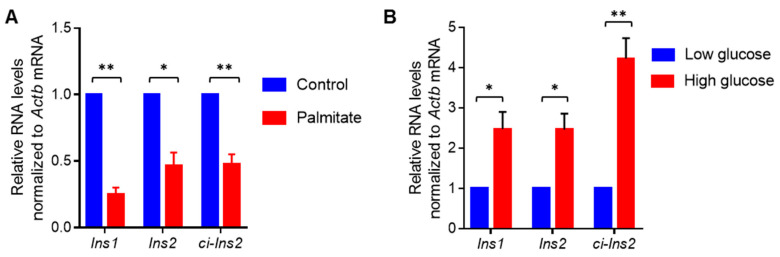
Altered expression of ci-Ins2 in βTC6 cells exposed to palmitate or high glucose. (**A**) RT-qPCR analysis of *Ins1*, *Ins2* and *ci-Ins2* in βTC6 cells cultured for 3 days in DMEM (25 mM glucose) + 1% fetal bovine serum (FBS) with or without 0.5 mM sodium palmitate. (**B**) RT-qPCR analysis of *Ins1, Ins2* and *ci-Ins2* in βTC6 cells cultured for 7 days in low glucose (2.5 mM) or high glucose (25 mM) containing DMEM supplemented with 15% FBS. All results represent means ± SEM from 4 independent experiments. * *p* < 0.05, ** *p* < 0.001.

The RNA-seq data suggested a decrease in *ci-Ins2* level in pancreatic islets from mice fed with HFD. Since long-term exposure to elevated glucose and/or fatty acids in the blood are known to impair pancreatic β-cell physiology during the development of diabetes, we wanted to investigate the impact of elevated levels of fatty acids and glucose on the expression of *ci-Ins2* in the βTC6 cells. Consistent with previous reports, palmitate treatment in the presence of high glucose reduced the expression of *Ins1* and *Ins2* mRNAs (Figure 5A) [38,39,40,41]. Furthermore, *ci-Ins2* expression was significantly reduced in palmitate-treated cells compared with the control-treated cells (Figure 5A). However, we did not evaluate the effect of palmitate on beta-cell apoptosis. Additionally, to study the effect of long-term exposure to high glucose on *ci-Ins2* levels, RT-qPCR analysis was performed in βTC6 cells cultured in low or high glucose conditions for 7 days. As shown in Figure 5B, *ci-Ins2* and the host gene *Ins2* mRNA were significantly upregulated in βTC6 cells cultured in the high glucose condition. However, further experiments are underway to find the regulatory effect of *ci-Ins2* on *Ins2* mRNA or *vice versa*.

### 2.6. Splicing Factors are Predicted to Interact with the Ins2 pre-mRNA and ci-Ins2

**Figure 6:**
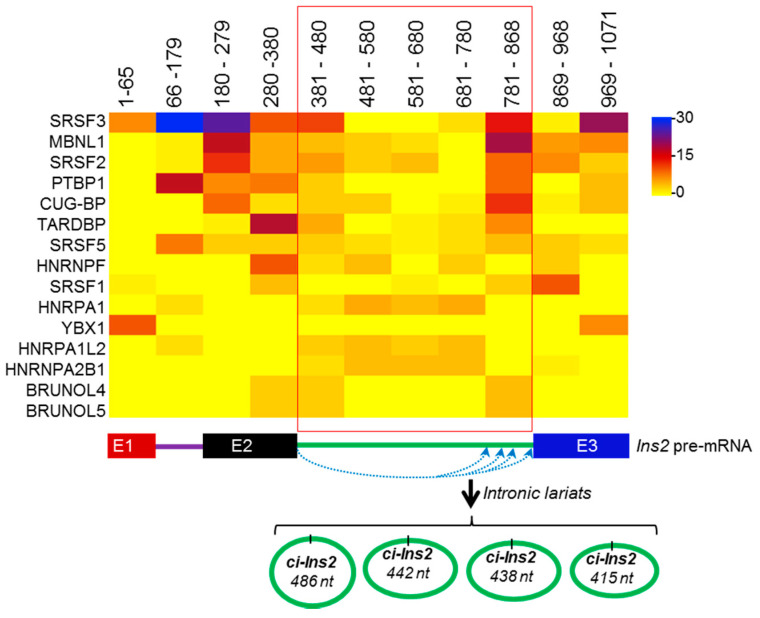
RNA-binding proteins (RBPs) predicted to associate with *ci-Ins2* and *Ins2* pre-mRNA. Heatmap of RBPs predicted by the beRBP algorithm to have ten or more number of binding sites on mouse *Ins2* pre-mRNA (*top*). The scale bar colors represent the number of binding sites on the specific region of *Ins2* pre-mRNA. Schematic representation of biogenesis of multiple ciRNAs from *Ins2* pre-mRNA (*bottom*). Straight lines and boxes represent introns and exons, respectively. The blue dotted arrows represent alternate intron lariat formation, generating multiple *ci-Ins2* transcripts.

It has been established that several RBPs modulate circRNA biogenesis, and circRNAs regulate the function of RBPs by acting as a decoy [10]. Prediction of RBPs interacting with pre-mRNA of *Ins2* using beRBP software has identified many splicing regulators, including SRSF3, PTBP1, CUG-BP, and MBNL1 (Figure 6, Table S5) [42]. The majority of the predicted proteins are not known to regulate *Ins2* mRNA splicing. However, their role in the *ci-Ins2* biogenesis warrants further investigation. Additionally, several RBPs, including SRSF3, PTBP1, CUG-BP, MBNL1, and TARDBP, were predicted to associate with *ci-Ins2,* suggesting that *ci-Ins2* might act as a sponge for these RBPs. Interestingly, PTBP1 and TARDBP (TDP-43) are known to be involved in insulin expression and secretion in pancreatic beta-cells [43,44]. However, dedicated efforts are underway to delineate the role of *ci-Ins2* in β-cells by acting as a decoy for any of these RBPs.

### 2.7. The ci-Ins2 May Regulate Beta-Cell Physiology by Sponging miRNAs

**Figure 7:**
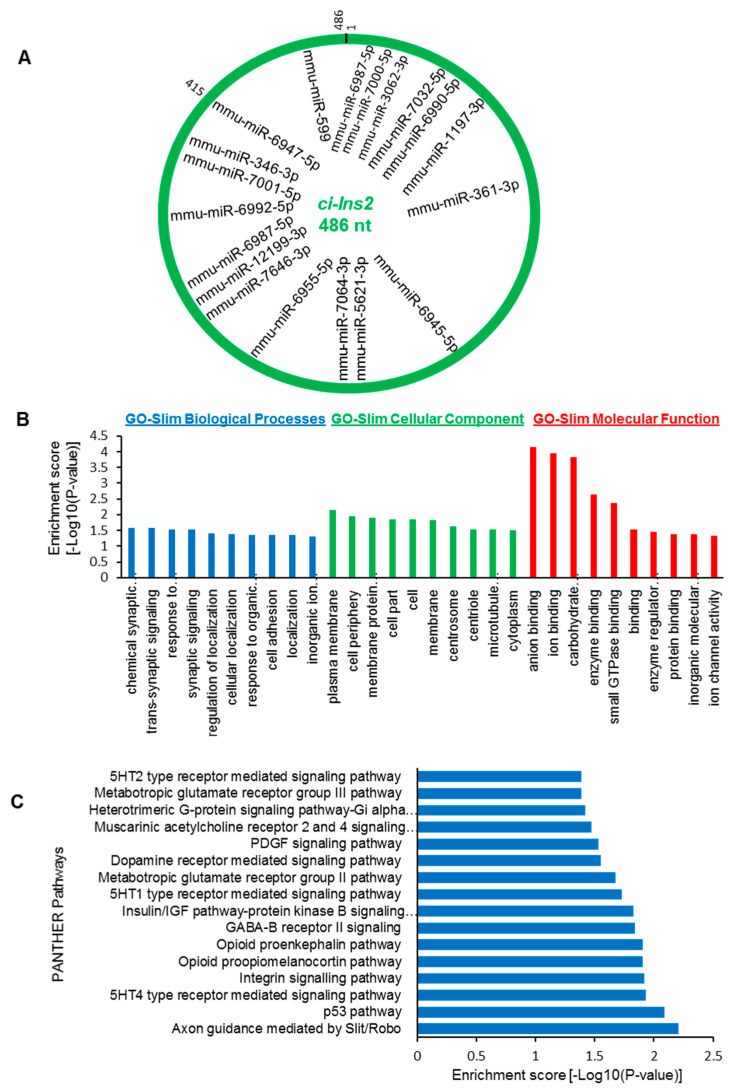
Prediction of *ci-Ins2*–miRNA–mRNA regulatory axis. (**A**) miRNAs predicted by miRDB to associate with *ci-Ins2* (486 nt). (**B**) The top 10 GO terms with the highest number of enriched genes for biological processes, cellular components, and molecular function for the mRNA targets of miRNAs associated with *ci-Ins2* (486 nt). (**C**) PANTHER pathways enriched for the mRNA targets of miRNAs associated with *ci-Ins2* (486 nt).

To investigate how *ci-Ins2* modulates β-cell physiology, we sought to identify *ci-Ins2* (486 nt) associated miRNAs. Interestingly, a number of miRNAs were predicted to interact with *ci-Ins2* using miRDB (Figure 7A) [45]. The majority of the miRNAs were found to be common for all four validated *ci-Ins2* transcripts. Furthermore, we identified downstream mRNAs targeted by the miRNAs associated with *ci-Ins2* using various software, including miRDB, RNA22, miRTarBase, and TargetScan [45,46,47,48]. The mRNAs predicted by two or more software and are differentially expressed in islets of mice fed with HFD compared to ND were further analyzed for GO and pathway enrichment (Table S6**) [49]**. GO-slim biological process analysis suggested that the target mRNAs were mainly enriched in chemical synaptic transmission, trans-synaptic signaling, response to endogenous stimulus, synaptic signaling, and regulation of localization and cellular localization. (Figure 7B). GO-slim cellular component analysis suggested the enrichment of various terms, including plasma membrane, cell periphery, membrane protein complex, cell part, cell, and membrane. Additionally, GO-slim molecular function analysis identified genes involved in anion binding, ion binding, carbohydrate derivative binding, enzyme binding, and small GTPase binding. (Figure 7B). Furthermore, pathway analysis of the target mRNAs using PANTHER identified several enriched pathways, including Axon guidance mediated by Slit/Robo, p53 pathway, 5HT4 type receptor mediated signaling pathway, integrin signaling pathway, opioid proopiomelanocortin pathway, GABA-B receptor II signaling, insulin/IGF pathway-protein kinase B signaling cascade, and PDGF signaling pathway. (Figure 7C) [49]. In sum, these data indicate that the circRNA–miRNA–mRNA axis may help us to explore the molecular mechanism of *ci-Ins2* in β-cell physiology.

## 3. Discussion

Recent developments in RNA-sequencing technologies have helped in exploring the characteristics of circRNAs across various tissues in various organisms [11]. The circRNAs have been shown to have tissue- and cell-line-specific expression. CircRNAs have been implicated in different pathophysiological conditions and regulate gene expression by acting as a sponge for RBPs and microRNAs, and competing with linear splicing [10]. However, the molecular mechanism of circRNA expression and their role in pancreatic β-cells is not understood completely [28].

Dysregulation of insulin production from pancreatic β-cells is one of the key causes of the development of diabetes [50,51]. Here, we explored the expression profile and the regulatory functions of circRNAs in β-cells. Recent studies have identified a large number of circRNAs in human and mouse pancreatic islets [32]. In this study, we used publicly available RNA-sequencing data to identify differentially expressed circRNAs in a diet-induced obesity mouse model. In this study, we detected >70,000 circRNAs in mouse islets, and several circRNAs were found to be dysregulated in the islets of mice fed with HFD compared to ND. Interestingly, three of the differentially expressed circRNAs were found to be generated from the mouse *Ins2* gene. In the mouse pancreatic islets, most genes generate one or a few circRNAs, supporting the notion that specific exons/introns are circularized in a regulated manner. By contrast, RNA-seq data indicated that *Ins2* pre-mRNA undergoes alternative backsplicing and generates multiple circRNAs. This data was further validated by RT–PCR with divergent primers placed on exon2 and intron2 of *Ins2* pre-mRNA, where multiple alternative circular transcripts were amplified (Figure 2). RT-PCR amplification of *circ-Ins2* resulted in a smear without a specific PCR product, and the level of expression was found to be very low in islets and βTC6 cells. Furthermore, RNA-seq data suggested the expression of 37 intronic *ci-Ins2* transcripts from intron 2 of *Ins2* pre-mRNA. However, PCR amplification of intron 2 of *Ins2* pre-mRNA using the divergent primers resulted in the amplification of a product corresponding to *ci-Ins2* with a length of 415 nt. Furthermore, RT-PCR analysis using the primer spanning the circRNA junction confirmed the expression of multiple circular intronic RNAs from intron2 that are collectively termed as *ci-Ins2,* with the longest one with 486 nt (Figure 4). In principle, the *ci-Ins2* RNAs are intron lariats generated by 2′–5′ end ligation during splicing [16,35]. However, we cannot exclude the possibility of some *ci-Ins2* RNAs with 3′–5′ end ligation. This observation supports the recent study that reported the use of multiple branchpoints during splicing in humans [37]. The usage of multiple branchpoints in *Ins2* pre-mRNA splicing may be advantageous to mutations and genetic variations and may be differentially regulated by specific splicing regulators. Although RNA-seq identified many intron-derived *ci-Ins2* RNAs, our PCR sequencing could validate the expression of only a few of them in βTC6 cells. The number of *ci-Ins2* RNAs identified in our PCR is far from complete as low abundant transcripts were most likely missed, and many of the lariat junctions differed by a few nucleotides, which is difficult to distinguish with primers spanning the junction sequence.

CircRNAs are resistant to exonuclease mediated degradation due to the lack of free ends [19,20,36,52]. As expected, *ci-Ins2* transcripts were found to be resistant to RNase R treatment. Furthermore, the junction sequences of *ci-Ins2* transcripts are similar to the previously published ci-RNA consensus sequence, suggesting that some of the *ci-Ins2* transcripts might be resistant to debranching (DBR) enzymes [16]. Since there are several transcripts of *ci-Ins2* generated due to alternate branchpoint selection and the huge amount of *Ins2* pre-mRNA transcription in β-cells, some of these lariats may be rapidly degraded by DBR enzymes. We hypothesize that *ci-Ins2* transcripts having a lariat junction sequence similar to the ci-RNA consensus sequence are resistant to DBR activity and remain stable (Figure S6B). However, further investigation is required to distinguish the stable *ci-Ins2* lariat population that is resistant to DBR, as well as to find whether the *ci-Ins2* transcripts contain 2′-5′ or 3′-5′ junctions.

It has been well established that long-term exposure of pancreatic β-cells to elevated levels of fatty acids and high glucose leads to impaired insulin transcription and secretion from pancreatic β-cells. Since we could not validate the altered expression of *ci-Ins2* in the HFD-fed animal models, we used the in vitro diabetic model, where the βTC6 were cultured in the presence of high concentration of palmitate and/or glucose [38,39,40]. Palmitate in the presence of high glucose is known to inhibit insulin transcription and secretion from β-cells [38,39,40]. As expected, our data suggested that palmitate in the presence of high glucose inhibited insulin gene expression and decreased *ci-Ins2* levels (Figure 5A). Additionally, glucose has been known to enhance insulin gene transcription and splicing [5,7]. Our data suggested that the levels of *ci-Ins2,* along with *Ins2* mRNA, were significantly upregulated in high-glucose treated cells compared to cells cultured in low glucose (Figure 5B). These data suggest that the altered expression of *ci-Ins2* could be associated with the levels of *Ins2* pre-mRNA transcription. Since ciRNAs are known to positively regulate transcription of their parent gene, the possibility of *Ins2* gene transcription regulation by *ci-Ins2* cannot be ruled out. However, further experiments are required to find the role of *ci-Ins2* on *Ins2* expression.

Previous work has indicated that inverted Alu sequences and RBPs regulate circRNA biogenesis [15,19,53]. To study the possible involvement of RBPs and splicing factors in the biogenesis of *ci-Ins2,* we analyzed the RBP binding sites in the *Ins2* pre-mRNA using the beRBP algorithm (Figure 6). This computational analysis identified several binding motifs for SRSF3, PTBP1, MBNL1, CUG-BP, SRSF5, and TARDBP on *Ins2* pre-mRNA, including intron2 that generates *ci-Ins2* (Figure 6). RBPs such as PTBP1 and TARDBP (TDP-43) have been shown to regulate insulin mRNA stability and early-stage insulin secretion, respectively [43,44]. We believe that altered expression of *ci-Ins2* possibly affects insulin expression and secretion from pancreatic β-cells by sponging PTBP1 and TARDBP. However, additional experiments are required to study the interaction of any of these RBPs with *ci-Ins2* and their role in insulin biosynthesis or secretion. Additionally, whether any of these RBPs are implicated in the alternative circularization of *Ins2* producing multiple *ci-Ins2* transcripts warrants further investigation. Since circRNAs are very well known to regulate gene expression by acting as miRNA sponges, we also analyzed the miRNAs associated with *ci-Ins2.* Our results suggest that *ci-Ins2* transcripts contain potential miRNA binding sites, indicating their potential to act as a decoy for the target miRNAs. Prediction of a circRNA–miRNA–mRNA regulatory network for *ci-Ins2* identified several genes that are involved in key biological processes and pathways known to regulate β-cell physiology (Figure 7). Additionally, the circRNA–miRNA–mRNA regulatory axis discovered here will provide a better understanding of the complex regulatory relationship and mechanism of the development of diabetes. Since these analyses were based on computational predictions, further experimental evidence is required to validate the target miRNA/mRNA expression and discover the underlying molecular mechanisms regulating β-cell physiology.

## 4. Materials and Methods

### 4.1. CircRNA Annotation

Circular RNAs were identified in publicly available RNA-sequencing data from pancreatic islets of mice fed with ND and HFD (accession GSE92602/PRJNA358100) [33]. Briefly, the raw RNA-seq reads were downloaded from SRA, and the quality was checked with FastQC. The reads were aligned to the mouse genome (mm10) using STAR aligner, and the alignment information was parsed using the CIRCexplorer2 parse module followed by identification and quantification of circRNAs using the CIRCexplorer2 annotation module [14]. Additionally, the Burrows–Wheeler aligner was used to align the reads with the mouse genome (mm10) followed by circRNA identification with CIRI2 (v 2.6) [54]. The differential expression of circRNAs was analyzed using the edgeR package [55].

### 4.2. Animals and Pancreatic Islet Isolation

Male C57Bl/6 mice with the age of 2–5 months were acquired from the Institute of Life Sciences breeding colonies. Pancreatic islets were isolated from the mice pancreas by collagenase digestion, followed by the Ficoll gradient separation method [56]. Briefly, the whole pancreas was dissected into small pieces in Hanks’ balanced salt solution (HBSS) and digested for 20 min at 37 °C with HBSS containing 0.5 mg/mL collagenase. The digested pancreas was washed with HBSS and layered on Ficoll (Sigma Aldrich) gradient, followed by centrifugation to isolate pancreatic islets. The isolated islets were washed with HBSS and were cultured in RPMI medium (Thermo Fisher Scientific) supplemented with 20% fetal bovine serum (FBS, Gibco) and antibiotics. All animal procedures were performed in agreement with the institutional animal ethics committee of the Institute of Life Sciences.

### 4.3. βTC6 Cell Culture and In Vitro Treatments

Mouse βTC6 cells were cultured in DMEM containing 15% FBS and antibiotics and were maintained in a 5% CO_2_ humidified atmosphere at 37 °C [56]. For glucose treatment experiments, βTC6 cells were cultured for 7 days in low glucose (2.5 mM) DMEM or high glucose (25 mM) DMEM supplemented with 15% FBS and antibiotics. Palmitate (100 mM) was prepared in 50% ethanol and FFA-free BSA (2 mM) was prepared in DMEM [40]. The palmitate solution was diluted 10 times with the BSA and incubated for 1 h at 37 °C to obtain the BSA-conjugated palmitate stock solution with 10 mM palmitate. For palmitate treatment, βTC6 cells cultured in high glucose (25 mM) DMEM supplemented with 1% FBS were treated with BSA-conjugated palmitate at a final concentration of 0.5 mM palmitate for 3 days. The control cells cultured in high glucose (25 mM) DMEM supplemented with 1% FBS were exposed to a vehicle containing the same amount of BSA as the BSA-conjugated palmitate solution.

### 4.4. RNA Isolation, RT-PCR, and Sanger Sequencing

Total RNA from pancreatic islets and βTC6 cells was isolated using TRIzol reagent, followed by reverse transcription (RT) with Maxima reverse transcriptase following the manufacturer’s protocol (Thermo Fisher Scientific). Specific primer sets (Table S1) were used for the PCR amplification of circRNAs and mRNAs using DreamTaq PCR Master Mix or PowerUp SYBR Green Master Mix, following the manufacturer’s instructions. PCR was set up with a cycle set up of 2 min at 95 °C followed by 35/40 cycles of 95 °C for 5 s, 60 °C for 20 s. RT-PCR products were resolved in SYBR-Gold-stained 2.5% agarose gels and visualized on an ultraviolet transilluminator. The RT-PCR products were purified and subjected to Sanger sequencing to check the specific amplification of the target circRNAs.

### 4.5. PCR Product Cloning and DNA Sequencing

RT-PCR products of *ci-Ins2* amplified with divergent primers were gel-purified using the PureLink Quick Gel Extraction Kit (Thermo Fisher Scientific). The purified PCR products were cloned into pGEM^®^-T Easy Vector Systems (Promega) following the manufacturer’s protocol. Individual positive clones were selected for culture, and plasmids were purified using the plasmid isolation kit (HiMedia). The sequences of the cloned PCR products were confirmed by Sanger sequencing of the plasmids with T7 primer.

### 4.6. Quantitative (q)PCR Analysis and RNase R Treatment

RT followed by quantitative PCR (RT-qPCR) analysis of target RNAs was performed using PowerUp SYBR Green Master Mix (Applied Biosystems) with specific primers (Table S1). RT-qPCR was performed on a QuantStudio 3/6 real-time PCR system (Thermo Fisher Scientific), and the relative RNA abundance was calculated using the delta-CT method. The circular nature of *ci-Ins2* and other circRNAs was tested using an RNase R treatment assay. Total RNA from βTC6 cells and islets was digested with 20 U of RNase R (Epicentre) at 37 °C for 30 min, followed by RT-qPCR analysis, as described previously [57].

### 4.7. Prediction of RBPs, miRNA, and mRNA Targets

The genomic sequences of *ci-Ins2* (486nt) and *Ins2* were retrieved from the UCSC genome browser (http://genome.ucsc.edu/). The RBPs associated with *Ins2* pre-mRNA were identified with the beRBP tool using the default parameters [42]. The number of RBP binding sites on the specific regions of the *Ins2* pre-mRNA is provided in Table S5. The miRNAs associated with *ci-Ins2* (486 nt) were identified using the miRDB web server (http://mirdb.org; accessed on 12 January 2020) [45]. Target mRNAs of miRNAs associated with *ci-Ins2* were identified using miRDB, RNA22, miRTarBase, and TargetScan software [45,46,47,48]. The differentially expressed mRNAs in islets of mice fed with HFD compared to ND were retrieved from the GEO data set (accession GSE92602) of the previous publication [33]. The mRNAs predicted by two or more software and differentially expressed in islets of mice fed with HFD compared to ND were selected for further analysis (Table S6). The Gene Ontology analysis for biological process, cellular component, and molecular function was performed, with a statistical overrepresentation test using Fisher’s extract and Bonferroni correction using the Panther database (http://www.pantherdb.org/; accessed on 4 April 2020) [49]. The pathway enrichment analysis was performed using the Panther database [49].

### 4.8. Statistical Analysis and Visualization

CircRNAs with log2 fold-changes greater than 2 and p-values less than 0.01 between ND- and HFD-fed mice islets were considered as differentially expressed. All experiments were repeated at least 3 times and were represented as the mean ± SEM. Comparison between control and treatment groups were analyzed with Student’s *t*-test and *p* < 0.05 was considered statistically significant. Heatmap for RBP binding sites and the differentially expressed circRNAs were generated in Excel and in R using ComplexHeatmap, respectively [58]. Graphs and plots were generated using Excel, GraphPad Prism, or ggplot2 package in R.

## 5. Conclusions

In summary, we have identified a number of DE-circRNAs in the islets of mice fed with HFD compared to ND. Our data discovered the expression of multiple *ci-Ins2* transcripts derived from intron2 of the mouse *Ins2* gene. The *ci-Ins2* transcripts may act as a decoy for RBPs and/or miRNAs due to the presence of RBP- and miRNA-binding sites, respectively. However, future studies analyzing the interaction of RBPs and miRNAs with *ci-Ins2* may uncover their role in β-cell physiology and the development of diabetes. As dysregulation of insulin biosynthesis is critical for the development of diabetes, interventions to modulate *Ins2* expression by regulating *ci-Ins2* function may be therapeutically valuable in the treatment of diabetes. Finally, a comprehensive understanding of the biogenesis and impact of *ci-Ins2* on β-cell gene expression programs will offer new avenues to explore the significance of circRNAs on β-cell proliferation, insulin biosynthesis, and insulin secretion.

## Figures and Tables

**Table 1:** List of differentially expressed circRNAs detected with CIRCexplorer2.

CircRNA_ID	Name of Isoform	Name of Gene	Splice Length	Type of CircRNA	logFC (HFD vs. ND)	*P* Value
chr16|20422304|20422485|R	NM_013790	Abcc5	181	circRNA	−2.9989	0.0044
chr1|155601453|155677234|F	NM_028250	Acbd6	310	circRNA	−3.0915	0.0027
chr1|82893582|82894474|F	NM_001347077	Agfg1	892	ciRNA	−3.6744	0.0026
chr1|177102961|177109738|R	NM_011785	Akt3	257	circRNA	2.3388	0.0035
chr1|177031649|177067333|R	NM_011785	Akt3	658	circRNA	−4.9995	0.0043
chr1|58059034|58065366|F	NM_009676	Aox1	797	circRNA	−4.7680	0.0096
chr13|94493668|94532066|F	NM_009680	Ap3b1	497	circRNA	5.1675	0.0023
chr9|44751215|44752275|R	NM_145985	Arcn1	314	circRNA	3.1211	0.0028
chr8|11781154|11785914|F	NM_001113517	Arhgef7	418	circRNA	3.3226	0.0009
chr12|101932967|101945919|R	NM_029705	Atxn3	638	circRNA	−3.7032	0.0024
chr2|59932104|59960105|R	NM_001001182	Baz2b	1064	circRNA	2.7952	0.0029
chr19|36986454|36992584|F	NM_001080706	Btaf1	1233	circRNA	3.8481	0.0012
chr8|124597498|124600487|F	NM_023709	Capn9	339	circRNA	−4.8492	0.0071
chr13|24164800|24178257|R	NM_001311122	Carmil1	233	circRNA	−3.4788	0.0061
chr10|41654941|41656332|R	NM_001358562	Ccdc162	423	circRNA	4.7722	0.0084
chr6|147507783|147562712|F	NM_001355714	Ccdc91	759	circRNA	−4.9599	0.0054
chr17|42805159|42830105|R	NM_009847	Cd2ap	1267	circRNA	−2.2265	0.0056
chr8|105643515|105666792|F	NM_001358924	Ctcf	1212	circRNA	4.7654	0.0098
chr9|3454539|3460131|F	NM_027545	Cwf19l2	567	circRNA	4.9208	0.0052
chr7|55873445|55875088|F	NM_001164661	Cyfip1	362	circRNA	−5.5388	0.0004
chr8|111010720|111011462|R	NM_001190800	Ddx19b	416	circRNA	−3.2670	0.0009
chr11|86784878|86793261|R	NM_026191	Dhx40	609	circRNA	3.6590	0.0035
chr4|99070177|99079849|R	NM_001290636	Dock7	646	circRNA	−2.9306	0.0070
chr14|66824344|66834376|R	NM_009955	Dpysl2	498	circRNA	−2.4682	0.0012
chr15|12878657|12890547|F	NM_001130149	Drosha	811	circRNA	−3.3908	0.0091
chr4|59690143|59691338|F	NM_153158	E130308A19Rik	1195	circRNA	2.9678	0.0070
chr4|58872588|58885498|R	NM_001355696	Ecpas	689	circRNA	4.7628	0.0099
chr18|33874141|33891476|R	NM_013512	Epb41l4a	591	circRNA	−2.0009	0.0013
chr11|26407547|26434500|F	NM_001277273	Fancl	267	circRNA	−4.7565	0.0099
chr9|78098345|78104354|R	NM_023605	Fbxo9	401	circRNA	4.8614	0.0061
chr6|99162838|99435345|R	NM_001347345	Foxp1	491	circRNA	−3.1755	0.0018
chr5|71623854|71642326|R	NM_001359041	Gabra4	929	circRNA	2.4293	0.0003
chr7|19164639|19164967|R	NM_001080815	Gipr	328	ciRNA	−4.9741	0.0046
chr6|86717385|86722665|R	NM_011818	Gmcl1	374	circRNA	−4.9008	0.0063
chr3|88880070|88887645|F	NM_001242372	Gon4l	390	circRNA	4.9467	0.0049
chr3|20058898|20076584|F	NM_001355097	Hltf	1131	circRNA	−4.8942	0.0064
chr16|4762558|4763846|F	NM_001136066	Hmox2	237	circRNA	3.1462	0.0029
chr6|51465178|51466222|R	NM_016806	Hnrnpa2b1	144	circRNA	−5.4402	0.0006
chr7|142678908|142679346|R	NM_001185083	Ins2	438	ciRNA	−2.6556	0.0050
chr7|142678860|142679346|R	NM_001185083	Ins2	486	ciRNA	−3.3804	0.0094
chr7|142678904|142679346|R	NM_001185083	Ins2	442	ciRNA	−3.3891	0.0090
chr13|44731712|44848421|F	NM_001205044	Jarid2	482	circRNA	5.2731	0.0012
chr4|149251740|149253751|R	NM_001290995	Kif1b	156	circRNA	−5.1133	0.0028
chr12|111785271|111785502|F	NM_001361611	Klc1	118	circRNA	−3.6522	0.0028
chr1|134475787|134485913|F	NM_001311136	Klhl12	418	circRNA	2.1707	0.0099
chr18|56739820|56743315|F	NM_010721	Lmnb1	552	circRNA	5.0791	0.0030
chr7|72161139|72185865|R	NM_001024703	Mctp2	503	circRNA	−5.0067	0.0042
chr4|87840681|87880148|R	NM_027326	Mllt3	935	circRNA	2.8933	0.0017
chr11|62419597|62423067|R	NM_001252313	Ncor1	376	circRNA	−2.0002	0.0045
chr8|61086398|61089799|F	NM_001293637	Nek1	418	circRNA	−2.9191	0.0065
chr5|24692806|24695590|F	NM_001305264	Nub1	301	circRNA	−4.7608	0.0098
chr2|121429453|121434093|F	NM_007952	Pdia3	665	circRNA	4.8197	0.0076
chr5|65663855|65666311|R	NM_001081321	Pds5a	389	circRNA	4.7741	0.0096
chr8|109876802|109895641|F	NM_001122594	Phlpp2	415	circRNA	−4.8009	0.0090
chr6|65862914|65901859|F	NM_027547	Prdm5	758	circRNA	−3.0054	0.0005
chr6|112665277|112681676|R	NM_001167730	Rad18	761	circRNA	2.0348	0.0004
chr17|65857661|65864732|R	NM_001198949	Ralbp1	1103	circRNA	3.1595	0.0024
chr4|135418379|135420419|R	NM_022980	Rcan3	346	circRNA	−2.1644	0.0079
chr5|63924734|63938033|R	NM_145923	Rell1	734	circRNA	2.1681	0.0009
chr17|29634660|29636022|F	NM_021419	Rnf8	313	circRNA	3.4555	0.0082
chr7|97616842|97653207|F	NM_001081267	Rsf1	546	circRNA	−5.4669	0.0005
chr3|130040673|130041447|R	NM_207209	Sec24b	774	circRNA	2.8977	0.0098
chr6|4707073|4719496|R	NM_001130188	Sgce	553	circRNA	−2.4581	0.0040
chr11|52236836|52243758|F	NM_011543	Skp1a	315	circRNA	−3.4104	0.0085
chr6|142101591|142133232|R	NM_023718	Slco1a6	936	circRNA	4.8197	0.0076
chr17|71455604|71465669|R	NM_028887	Smchd1	452	circRNA	−5.4092	0.0007
chr1|192930477|192986882|R	NM_001301370	Syt14	1103	circRNA	2.4311	0.0021
chr10|56087530|56089839|R	NM_001033385	Tbc1d32	326	circRNA	−4.8798	0.0066
chr11|121602987|121611748|F	NM_029878	Tbcd	871	circRNA	−5.4350	0.0007
chr1|135304429|135309198|R	NM_001360857	Timm17a	304	circRNA	−2.2046	0.0012
chr1|37783816|37811027|R	NM_207228	Tsga10	732	circRNA	4.9306	0.0051
chr5|92166530|92167333|F	NM_019490	Uso1	260	circRNA	4.8256	0.0075
chr11|85077264|85083894|R	NM_001029934	Usp32	385	circRNA	−3.3658	0.0095
chr11|23333438|23345217|F	NM_001190401	Usp34	690	circRNA	4.8640	0.0069
chr7|99065799|99099473|R	NM_178635	Uvrag	320	circRNA	−5.6041	0.0002
chr3|108618489|108638757|F	NM_001358053	Wdr47	2071	circRNA	−4.0332	0.0003
chr8|107483331|107485642|F	NM_025830	Wwp2	235	circRNA	−3.4087	0.0083

## Supplementary Materials

The following are available online at https://www.mdpi.com/1422-0067/21/12/4302/s1. Figure S1: Characteristics of circRNAs in mouse pancreatic islets. Figure S2: Selected list of circRNAs from RNA-seq data. Figure S3: Validation of circRNAs in βTC6 cells. Figure S4: Validation of circular Ins2 transcripts in islets. Figure S5: Lariat branchpoint sequence of ci-Ins2_415. Figure S6: Sequence of ci-Ins2 transcripts. Table S1: List of oligos used for the study. Table S2: circRNA annotation by CIRCexplorer2. Table S3: circRNA annotation by CIRI2. Table S4: List of circRNAs derived from mouse Ins1 and Ins2 gene detected using CIRCexplorer2. Table S5: List of RBPs and the number of binding sites predicted in different regions of mouse Ins2 pre-mRNA using beRBP. Table S6: CircRNA–miRNA–mRNA regulatory network for ci-Ins2 (486 nt).
