# Supplementary material for: Identification and Characterization of Circular Intronic RNAs Derived from Insulin Gene
Source: Int J Mol Sci. 2020 Jun 17;21(12):4302. doi: 10.3390/ijms21124302 (PMC7352490; doi:10.3390/ijms21124302)
Supplement: Supplementary file 1 [file ijms-21-04302-s001.zip › Das et al_Supplementary/Das et al. Supplementary Figures_Tables.pdf]

# Identification and characterization of circular intronic RNAs derived from insulin gene

**Debojyoti Das[1,2,#], Aniruddha Das[1,2,#], Mousumi Sahu[1], Smruti Sambhav Mishra[1,] Shaheerah Khan[1], Pruthvi R. Bejugam[1], Pranita K. Rout[1], Arundhati Das[1,2], Shehnaz Bano[3], Gyan Prakash Mishra[1], Sunil K. Raghav[1], Anshuman Dixit[1], and Amaresh C. Panda[1,*]**

[1] Institute of Life Sciences (ILS), Nalco Square, Bhubaneswar, Odisha, India;
[2] School of Biotechnology, KIIT University, Bhubaneswar, Odisha, India
[3] National Center for Cell Sciences (NCCS), Pune, Maharashtra, India
[#] Contributed equally to this work

* Correspondence: Amaresh C Panda, Email: amaresh.panda@ils.res.in, Tel: +91 674-2304314

**Figure S1**

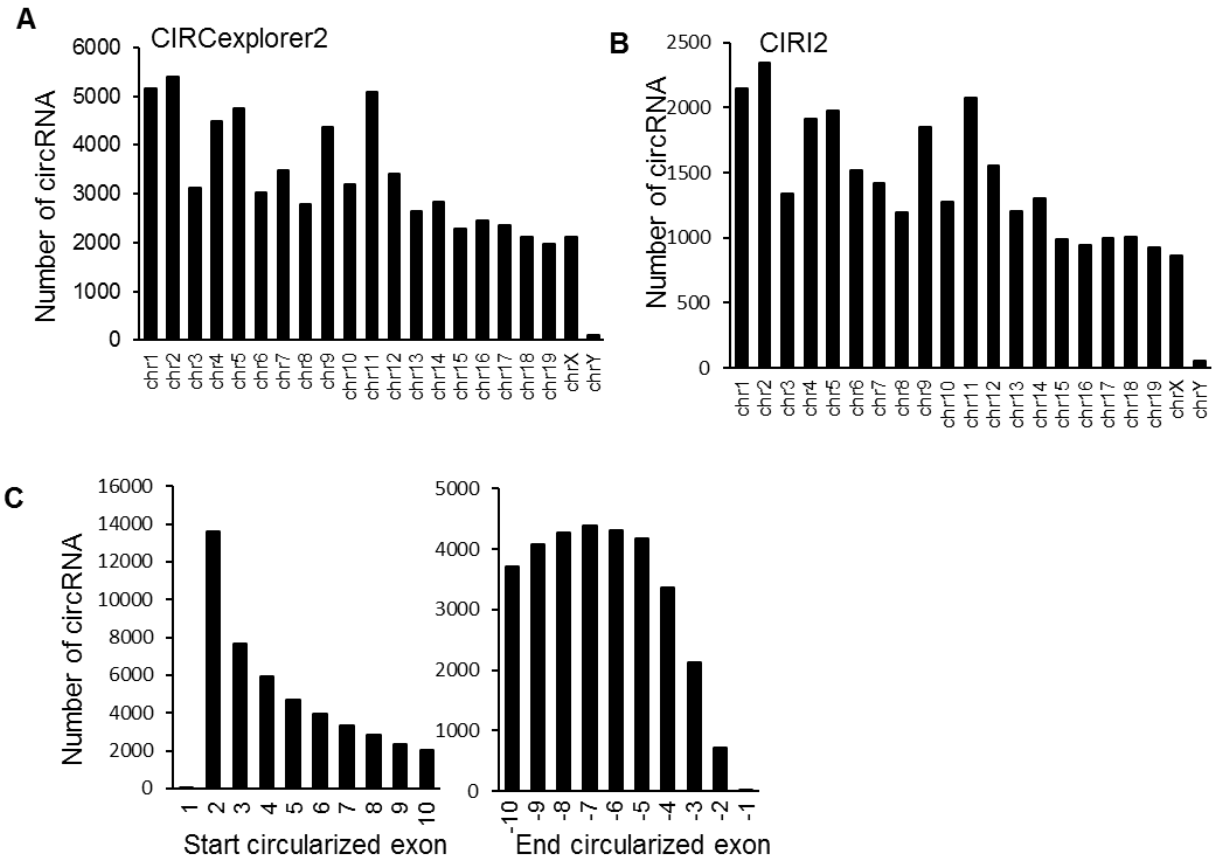


**Supplementary Figure S1: Characteristics of circRNAs in mouse pancreatic islets**. **(A)** Distribution of circRNAs per chromosome detected with CIRCexplorer2. **(B)** Distribution of circRNAs per chromosome detected with CIRI2. **(C)** Distribution of the start (*left*) and the end (*right*) 10 exons of linear RNAs used for the biogenesis of exonic circRNAs detected with CIRCexplorer2.

**Figure S2**

**A**

| circRNA Name | Parental gene | circRNA_ID | Splice length | circRNA type | logFC (HFD/ND) | Pvalue |
|---|---|---|---|---|---|---|
| ci-Ins2-438 | Ins2 | chr7\|142678908\|142679346\|R | 438 | ciRNA | -2.655639 | 0.004973 |
| ci-Ins2-442 | Ins2 | chr7\|142678904\|142679346\|R | 442 | ciRNA | -3.389091 | 0.008988 |
| ci-Ins2-486 | Ins2 | chr7\|142678860\|142679346\|R | 486 | ciRNA | -3.380421 | 0.009369 |
| ci-Ins2-415 | Ins2 | chr7\|142678931\|142679346\|R | 415 | ciRNA | -2.5677 | 0.2984 |
| circBckdhb | Bckdhb | chr9\|83988775\|84012619\|F | 608 | circRNA | 0.589953 | 0.0001 |
| circGlis3 | Glis3 | chr19\|28530872\|28531986\|R | 1114 | circRNA | 0.032029 | 0.8302 |
| circCdk8 | Cdk8 | chr5\|146259504\|146271735\|F | 328 | circRNA | -0.42013 | 0.0256 |
| circAmbra1 | Ambra1 | chr2\|91805023\|91810209\|F | 267 | circRNA | -1.039486 | 0.0072 |
| circAsph | Asph | chr4\|9635900\|9639347\|R | 219 | circRNA | 0.351325 | 0.0087 |
| circNbas | Nbas | chr12\|13482385\|13484044\|F | 586 | circRNA | 0.4631983 | 0.00542 |

**B**

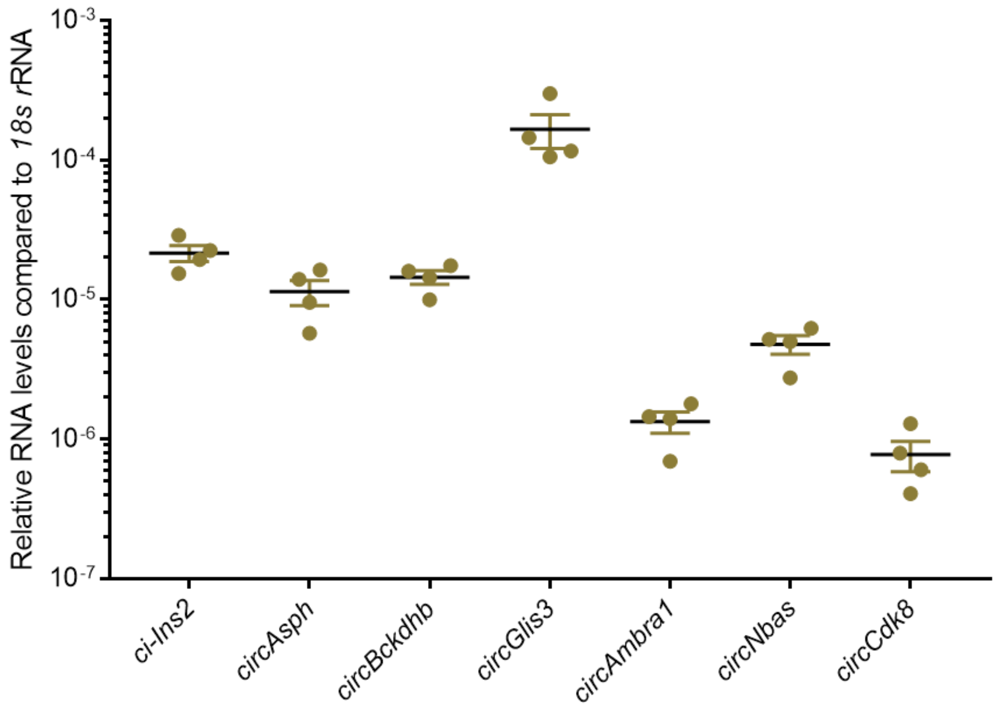


**Supplementary Figure S2: Selected list of circRNAs from RNA-seq data**. (**A)** A list of validated circRNAs detected using CIRCexplorer2. (**B**) Expression levels of selected circRNAs in βTC6 cells compared to *18s* rRNA using RT-qPCR analysis.

**Figure S3**

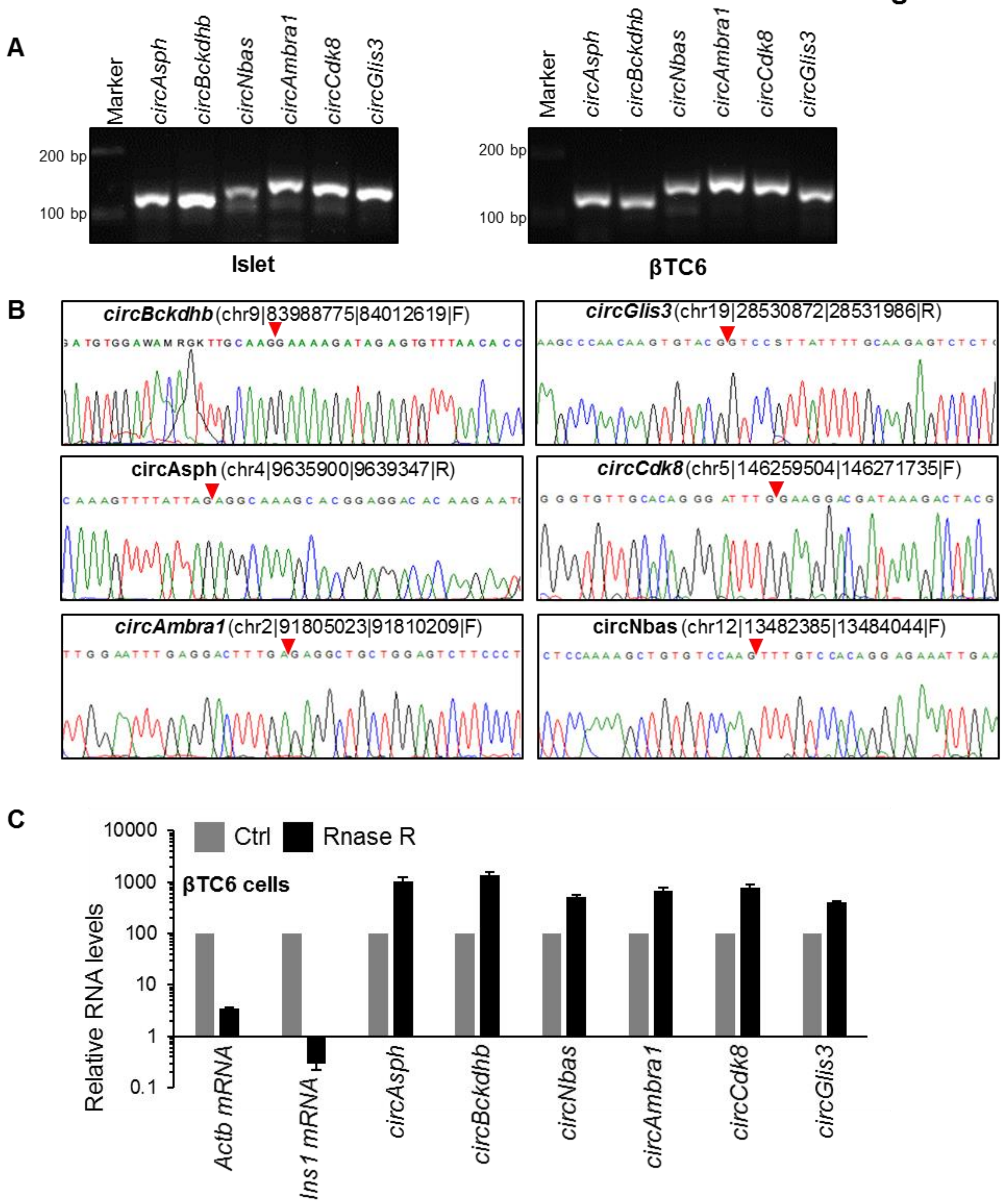


**Supplementary Figure S3: Validation of circRNAs in βTC6 cells**. (**A)** The RT-PCR results of selected circRNAs amplified from mice pancreatic islets (*left*) and βTC6 (*right*) with divergent primers and visualized in SYBR Gold stained 2.5 % agarose gel. (**B)** RT-PCR products were purified and sequenced to confirm the junction sequences of detected circRNAs from βTC6 cells. (**C)** RT-qPCR analysis showing the levels of mRNAs and circRNAs in βTC6 cells upon RNase R treatment. Data represents the means ± S.E.M. from three independent experiments.

**Figure S4**

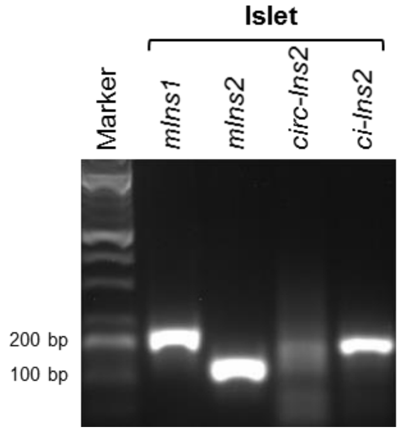


**Supplementary Figure S4: Validation of circular *Ins2* transcripts in islets.** The RT-PCR products of *Ins1*, *Ins2*, *circ-Ins2*, and *ci-Ins2* in mouse pancreatic islets were resolved and visualized in SYBR Gold stained 2.5 % agarose gel.

**Figure S5**

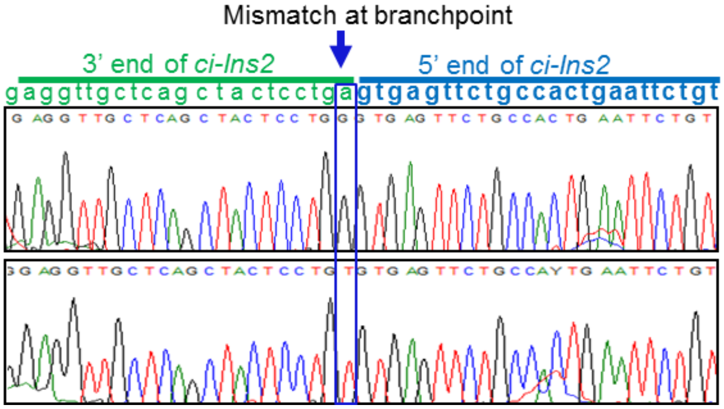


***ci-Ins2_415*** (415 nt; chr7|142678931|142679346|R)

**Supplementary Figure S5: Lariat branch point sequence of *ci-Ins2_415*.** PCR products of *ci-Ins2* from βTC6 cells were purified and cloned into pGEM®-T Easy vector followed sequencing to confirm the branchpoint junction sequences of *ci-Ins2* transcripts. Majority of the clones correspond to the lariat sequence of ci-Ins2 (415 nt). The blue box in the chromatograph shows sequence mismatch error (G/T) at the branchpoint nucleotide-A.

## Figure S6

**A**

**<u>*ci-Ins2* (486 nt; chr7|142678860|142679346|R)</u>**

gtgagttctgccactgaattctgtccccagtgctaactaccctggttttcttcacacttgggacattgtaaattgtgtccta
ggtgtggagggtctcgggataaccagggagtggggacacgtttctgggggaagctagacatatgtaaacatggcagctgcca
ggaatgagtaagaatcctgccttaaggggtccttggtggtagtaacttgggacatgtgactagatcccaggataggtaccta
tttagggccctcatagagcactgcactgactgaagatgagtaggctttagaggcccatgtgtccatccatgaccagtgactt
gtcccacaggcatgcaacccctgccacctgcaggggttaaggggcgagaaaacctggggtagtaggaggttgctcagctact
cctgactggattttcctatgtgtctttgcttctgtgctgctgatgccctggcctgctctgacacaacctccctggc

**<u>*ci-Ins2* (442 nt; chr7|142678904|142679346|R)</u>**

gtgagttctgccactgaattctgtccccagtgctaactaccctggttttcttcacacttgggacattgtaaattgtgtccta
ggtgtggagggtctcgggataaccagggagtggggacacgtttctgggggaagctagacatatgtaaacatggcagctgcca
ggaatgagtaagaatcctgccttaaggggtccttggtggtagtaacttgggacatgtgactagatcccaggataggtaccta
tttagggccctcatagagcactgcactgactgaagatgagtaggctttagaggcccatgtgtccatccatgaccagtgactt
gtcccacaggcatgcaacccctgccacctgcaggggttaaggggcgagaaaacctggggtagtaggaggttgctcagctact
cctgactggattttcctatgtgtctttgcttc

**<u>*ci-Ins2* (438 nt; chr7|142678908|142679346|R)</u>**

gtgagttctgccactgaattctgtccccagtgctaactaccctggttttcttcacacttgggacattgtaaattgtgtccta
ggtgtggagggtctcgggataaccagggagtggggacacgtttctgggggaagctagacatatgtaaacatggcagctgcca
ggaatgagtaagaatcctgccttaaggggtccttggtggtagtaacttgggacatgtgactagatcccaggataggtaccta
tttagggccctcatagagcactgcactgactgaagatgagtaggctttagaggcccatgtgtccatccatgaccagtgactt
gtcccacaggcatgcaacccctgccacctgcaggggttaaggggcgagaaaacctggggtagtaggaggttgctcagctact
cctgactggattttcctatgtgtctttg

**<u>*ci-Ins2* (415 nt; chr7|142678931|142679346|R)</u>**

gtgagttctgccactgaattctgtccccagtgctaactaccctggttttcttcacacttgggacattgtaaattgtgtccta
ggtgtggagggtctcgggataaccagggagtggggacacgtttctgggggaagctagacatatgtaaacatggcagctgcca
ggaatgagtaagaatcctgccttaaggggtccttggtggtagtaacttgggacatgtgactagatcccaggataggtaccta
tttagggccctcatagagcactgcactgactgaagatgagtaggctttagaggcccatgtgtccatccatgaccagtgactt
gtcccacaggcatgcaacccctgccacctgcaggggttaaggggcgagaaaacctggggtagtaggaggttgctcagctact
cctga

**B**

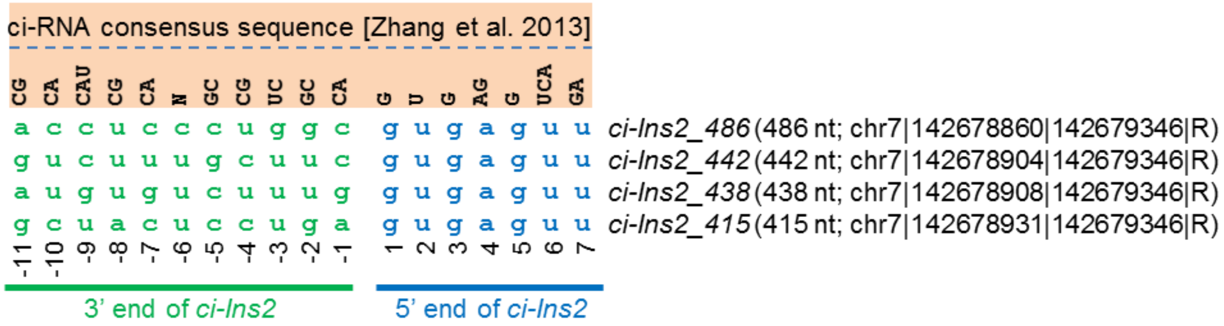


**Supplementary Figure S6. Sequence of *ci-Ins2* transcripts. (A)** Sequences of validated *ci-Ins2* transcripts generated from the intron 2 of *Ins2* pre-mRNA. (**B**) Representations of the lariat junction sequence (11 nt from the end and 7 nt from the start) of validated *ci-Ins2* RNAs and the ci-RNA consensus sequence reported by *Zhang et al. 2013* [Reference 16].

**Supplementary Table S1:** List of oligos used for the study

| Oligo name | Sequence (5'-3') |
|---|---|
| 18S rRNA-F | GTAACCCGTTGAACCCCATT |
| 18S rRNA-R | CCATCCAATCGGTAGTAGCG |
| Gapdh-F | AACTTTGGCATTGTGGAAGG |
| Gapdh-R | GGATGCAGGGATGATGTTCT |
| mActb-F | TGTTACCAACTGGGACGACA |
| mActb-R | GGGGTGTTGAAGGTCTCAAA |
| mIns1-F | AAGCAGGTCATTGTTTCAACA |
| mIns1-R | TTGGGTGTGTAGAAGAAGCC |
| mIns2-R | CAAAGGTGCTGCTTGACAAA |
| mIns2-F | CAGCAAGCAGGAAGGTTATT |
| circ-Ins2-div-F | TGGAGGCTCTCTACCTGGTG |
| circ-Ins2-div-R | CAGGAAGCGCATCCACAG |
| ci-Ins2-div-F | ACCTGCAGGGGTTAAGGGGCGA |
| ci-Ins2-div-R | AGACCCTCCACACCTAGGACAC |
| ciIns2_415_junc-F | CTACTCCTGAGTGAGTTCTG |
| ciIns2_438_junc-F | TGTGTCTTTGGTGAGTTCTG |
| ciIns2_442_junc-F | GTCTTTGCTTCGTGAGTTCTG |
| ci-Ins2 486_junc-F | CCTCCCTGGCGTGAGTTCTG |
| ci-Ins2-common-R | CCCCTTAAGGCAGGATTCTT |
| circAsph_F | GGCGATGGAGACTTTGATGT |
| circAsph_R | GCAATGCAATGACCATGAAC |
| circBckdhb_F | GTGCCTTGGGATGTGGATAC |
| circBckdhb_R | TTCCGCAATAGCTGTAGCAC |
| circGlis3_F | GGACTGGCTGCCCTAGAAG |
| circGlis3_R | TCAAAGTCGTGGACACCAGA |
| circNbas_F | CCTGTGATGTCTGCCTGAAG |
| circNbas_R | CAAAGAGGTGAAGGGTTTGG |
| CircCdk8_F | GGGGAATGGTGAAGTCACTG |
| circCdk8_R | CTGCATGCCGACATAGAAAT |
| circAmbra1_F | CAGAATCGGCTTCGTTCTTC |
| circAmbra1_R | GTGCTCTCTGGATCCTGCTC |

**Supplementary Table S2:** circRNA annotation by CIRCexplorer2.

**Supplementary Table S3:** circRNA annotation by CIRI2.

**Supplementary Table S4:** List of circRNAs derived from mouse *Ins1* and *Ins2* gene detected using CIRCexplorer2. The Bold and highlighted circRNAs were PCR validated in βTC6 cells.

| circRNA_ID | Splice length | Type of circular RNA | Name of gene | Name of isoform | logFC (HFD vs ND) | PValue |
|---|---|---|---|---|---|---|
| **chr7\|142678860\|142679346\|R** | **486** | **ciRNA** | **Ins2** | **NM_001185083** | **-3.3804** | **0.0094** |
| chr7\|142678861\|142679346\|R | 485 | ciRNA | Ins2 | NM_001185083 | -1.8602 | 0.0115 |
| chr7\|142678862\|142679346\|R | 484 | ciRNA | Ins2 | NM_001185083 | -0.0678 | 0.8479 |
| chr7\|142678863\|142679346\|R | 483 | ciRNA | Ins2 | NM_001185083 | -3.1554 | 0.1642 |
| chr7\|142678864\|142679346\|R | 482 | ciRNA | Ins2 | NM_001185083 | -1.5410 | 0.4156 |
| chr7\|142678865\|142679346\|R | 481 | ciRNA | Ins2 | NM_001185083 | -0.7983 | 0.6450 |
| chr7\|142678866\|142679346\|R | 480 | ciRNA | Ins2 | NM_001185083 | -1.8518 | 0.3481 |
| chr7\|142678867\|142679346\|R | 479 | ciRNA | Ins2 | NM_001185083 | -0.6998 | 0.7369 |
| chr7\|142678886\|142679346\|R | 460 | ciRNA | Ins2 | NM_001185083 | 1.9570 | 0.3227 |
| chr7\|142678894\|142679346\|R | 452 | ciRNA | Ins2 | NM_001185083 | -2.6842 | 0.2809 |
| chr7\|142678897\|142679346\|R | 449 | ciRNA | Ins2 | NM_001185083 | 0.1052 | 0.9676 |
| chr7\|142678900\|142679346\|R | 446 | ciRNA | Ins2 | NM_001185083 | -1.8518 | 0.3481 |
| chr7\|142678901\|142679346\|R | 445 | ciRNA | Ins2 | NM_001185083 | -2.5677 | 0.2984 |
| chr7\|142678902\|142679346\|R | 444 | ciRNA | Ins2 | NM_001185083 | 0.9340 | 0.1620 |
| chr7\|142678903\|142679346\|R | 443 | ciRNA | Ins2 | NM_001185083 | -1.5621 | 0.4099 |
| **chr7\|142678904\|142679346\|R** | **442** | **ciRNA** | **Ins2** | **NM_001185083** | **-3.3891** | **0.0090** |
| chr7\|142678905\|142679346\|R | 441 | ciRNA | Ins2 | NM_001185083 | -0.5298 | 0.2251 |
| chr7\|142678906\|142679346\|R | 440 | ciRNA | Ins2 | NM_001185083 | -0.7810 | 0.3636 |
| chr7\|142678907\|142679346\|R | 439 | ciRNA | Ins2 | NM_001185083 | -0.8397 | 0.3184 |
| **chr7\|142678908\|142679346\|R** | **438** | **ciRNA** | **Ins2** | **NM_001185083** | **-2.6556** | **0.0050** |
| chr7\|142678909\|142679346\|R | 437 | ciRNA | Ins2 | NM_001185083 | -0.0175 | 0.9803 |
| chr7\|142678910\|142679346\|R | 436 | ciRNA | Ins2 | NM_001185083 | 0.5333 | 0.2613 |
| chr7\|142678911\|142679346\|R | 435 | ciRNA | Ins2 | NM_001185083 | -1.0172 | 0.0185 |
| chr7\|142678912\|142679346\|R | 434 | ciRNA | Ins2 | NM_001185083 | 0.8520 | 0.6798 |
| chr7\|142678913\|142679346\|R | 433 | ciRNA | Ins2 | NM_001185083 | 0.4149 | 0.7938 |
| chr7\|142678914\|142679346\|R | 432 | ciRNA | Ins2 | NM_001185083 | -3.0570 | 0.1781 |
| chr7\|142678915\|142679346\|R | 431 | ciRNA | Ins2 | NM_001185083 | -1.1527 | 0.4816 |
| chr7\|142678917\|142679346\|R | 429 | ciRNA | Ins2 | NM_001185083 | -1.8068 | 0.3539 |
| chr7\|142678918\|142679346\|R | 428 | ciRNA | Ins2 | NM_001185083 | 3.4918 | 0.1026 |
| chr7\|142678919\|142679346\|R | 427 | ciRNA | Ins2 | NM_001185083 | 0.1028 | 0.9682 |
| chr7\|142678920\|142679346\|R | 426 | ciRNA | Ins2 | NM_001185083 | 0.0780 | 0.9671 |
| **chr7\|142678931\|142679346\|R** | **415** | **ciRNA** | **Ins2** | **NM_001185083** | **-2.5677** | **0.2984** |
| chr7\|142678933\|142679346\|R | 413 | ciRNA | Ins2 | NM_001185083 | 0.0612 | 0.9793 |
| chr7\|142678935\|142679346\|R | 411 | ciRNA | Ins2 | NM_001185083 | -1.8068 | 0.3539 |
| chr7\|142678938\|142679346\|R | 408 | ciRNA | Ins2 | NM_001185083 | -1.8784 | 0.3448 |
| chr7\|142678964\|142679346\|R | 382 | ciRNA | Ins2 | NM_001185083 | -2.5677 | 0.2984 |
| chr7\|142678965\|142679346\|R | 381 | ciRNA | Ins2 | NM_001185083 | -1.8518 | 0.3481 |
| chr7\|142679346\|142679547\|R | 201 | circRNA | Ins2 | NM_001185083 | 0.3601 | 0.7964 |
| chr7\|142679346\|142679559\|R | 213 | circRNA | Ins2 | NM_008387 | -1.8518 | 0.3481 |
| chr19\|52264491\|52264609\|F | 118 | ciRNA | Ins1 | NM_008386 | -1.8784 | 0.3448 |
| chr19\|52264491\|52264593\|F | 102 | ciRNA | Ins1 | NM_008386 | -3.4188 | 0.1163 |

**Supplementary Table S5:** List of RBPs and the number of binding sites predicted on different regions of mouse *Ins2* pre-mRNA using beRBP.

| | **Exon1** | **Intron1** | **Exon2** | | **Intron2** | | | | | **Exon3** | |
|---|---|---|---|---|---|---|---|---|---|---|---|
| | **1-65** | **66-179** | **180-279** | **280-380** | **381-480** | **481-580** | **581-680** | **681-780** | **781-868** | **869-968** | **969-1071** |
| **SRSF3** | 7 | 29 | 24 | 10 | 11 | 0 | 0 | 2 | 14 | 1 | 20 |
| **MBNL1** | 0 | 1 | 17 | 5 | 4 | 3 | 2 | 0 | 19 | 6 | 7 |
| **SRSF2** | 0 | 1 | 12 | 5 | 6 | 3 | 4 | 0 | 9 | 7 | 3 |
| **PTBP1** | 0 | 17 | 7 | 8 | 3 | 0 | 0 | 0 | 9 | 0 | 4 |
| **CUG-BP** | 0 | 0 | 9 | 2 | 3 | 3 | 0 | 1 | 12 | 1 | 4 |
| **TARDBP** | 0 | 0 | 1 | 18 | 5 | 0 | 1 | 2 | 7 | 0 | 0 |
| **SRSF5** | 0 | 8 | 3 | 3 | 3 | 2 | 1 | 2 | 4 | 3 | 2 |
| **HNRNPF** | 0 | 0 | 0 | 10 | 2 | 4 | 0 | 3 | 0 | 3 | 0 |
| **SRSF1** | 1 | 0 | 0 | 4 | 0 | 0 | 1 | 0 | 3 | 10 | 0 |
| **HNRPA1** | 0 | 2 | 0 | 0 | 2 | 5 | 4 | 5 | 0 | 0 | 0 |
| **YBX1** | 10 | 0 | 0 | 0 | 0 | 0 | 0 | 0 | 0 | 0 | 7 |
| **HNRPA1L2** | 0 | 2 | 0 | 0 | 3 | 4 | 3 | 4 | 0 | 0 | 0 |
| **HNRNPA2B1** | 0 | 0 | 0 | 0 | 2 | 4 | 4 | 4 | 0 | 1 | 0 |
| **BRUNOL4** | 0 | 0 | 0 | 3 | 3 | 0 | 0 | 0 | 4 | 0 | 0 |
| **BRUNOL5** | 0 | 0 | 0 | 3 | 3 | 0 | 0 | 0 | 4 | 0 | 0 |
| **HNRNPK** | 0 | 2 | 4 | 0 | 0 | 0 | 0 | 0 | 0 | 0 | 3 |
| **PCBP1** | 0 | 2 | 5 | 0 | 0 | 0 | 0 | 0 | 0 | 0 | 2 |
| **RBM38** | 0 | 0 | 0 | 3 | 3 | 1 | 0 | 0 | 2 | 0 | 0 |
| **TRA2B** | 0 | 0 | 0 | 0 | 4 | 1 | 0 | 1 | 0 | 2 | 0 |
| **YBX2** | 3 | 0 | 0 | 0 | 0 | 0 | 0 | 0 | 2 | 1 | 2 |
| **HNRNPH2** | 0 | 0 | 0 | 2 | 1 | 2 | 0 | 0 | 0 | 2 | 0 |
| **RBM24** | 0 | 0 | 0 | 3 | 2 | 0 | 0 | 0 | 2 | 0 | 0 |
| **SRSF7** | 0 | 0 | 0 | 0 | 0 | 0 | 4 | 0 | 0 | 0 | 3 |
| **BRUNOL6** | 0 | 0 | 0 | 4 | 2 | 0 | 0 | 0 | 0 | 0 | 0 |
| **HNRNPL** | 0 | 0 | 0 | 3 | 0 | 0 | 0 | 0 | 2 | 1 | 0 |
| **HNRPLL** | 0 | 0 | 0 | 3 | 0 | 0 | 0 | 3 | 0 | 0 | 0 |
| **PCBP2** | 0 | 0 | 4 | 0 | 0 | 0 | 0 | 0 | 1 | 0 | 1 |
| **PCBP3** | 0 | 3 | 0 | 0 | 0 | 0 | 0 | 0 | 3 | 0 | 0 |
| **CPEB4** | 0 | 0 | 2 | 1 | 2 | 0 | 0 | 0 | 0 | 0 | 0 |
| **DAZAP1** | 0 | 2 | 0 | 0 | 0 | 0 | 1 | 2 | 0 | 0 | 0 |
| **ESRP2** | 0 | 0 | 0 | 0 | 1 | 3 | 1 | 0 | 0 | 0 | 0 |
| **HuR** | 0 | 0 | 0 | 0 | 2 | 0 | 0 | 0 | 3 | 0 | 0 |
| **IGF2BP3** | 3 | 0 | 0 | 2 | 0 | 0 | 0 | 0 | 0 | 0 | 0 |
| **PABPC3** | 3 | 0 | 0 | 0 | 0 | 0 | 0 | 1 | 0 | 0 | 1 |
| **PABPC4** | 3 | 0 | 0 | 0 | 0 | 0 | 0 | 0 | 0 | 0 | 2 |
| **RBM28** | 0 | 0 | 0 | 0 | 1 | 0 | 0 | 2 | 0 | 2 | 0 |
| **HNRNPC** | 0 | 0 | 2 | 0 | 2 | 0 | 0 | 0 | 0 | 0 | 0 |
| **IGF2BP2** | 3 | 0 | 0 | 1 | 0 | 0 | 0 | 0 | 0 | 0 | 0 |
| **NOVA1** | 0 | 0 | 0 | 0 | 0 | 0 | 0 | 4 | 0 | 0 | 0 |
| **PABPC1** | 2 | 0 | 0 | 0 | 0 | 0 | 0 | 0 | 0 | 0 | 2 |
| **SART3** | 2 | 0 | 0 | 0 | 0 | 0 | 0 | 0 | 0 | 0 | 2 |
| **TIAI** | 0 | 0 | 2 | 1 | 1 | 0 | 0 | 0 | 0 | 0 | 0 |

| | | | | | | | | | | | |
|---|---|---|---|---|---|---|---|---|---|---|---|
| **U2AF2** | 0 | 0 | 0 | 0 | 3 | 0 | 0 | 0 | 1 | 0 | 0 |
| **ZC3H14** | 0 | 0 | 0 | 0 | 2 | 0 | 0 | 0 | 2 | 0 | 0 |
| **FMR1** | 0 | 0 | 0 | 0 | 0 | 0 | 3 | 0 | 0 | 0 | 0 |
| **HNRNPH1** | 0 | 0 | 0 | 1 | 0 | 0 | 2 | 0 | 0 | 0 | 0 |
| **LIN28A** | 0 | 0 | 0 | 0 | 1 | 0 | 0 | 1 | 0 | 1 | 0 |
| **RALY** | 0 | 0 | 1 | 0 | 2 | 0 | 0 | 0 | 0 | 0 | 0 |
| **RBM42** | 0 | 0 | 0 | 0 | 0 | 0 | 0 | 0 | 0 | 0 | 3 |
| **RBM46** | 1 | 0 | 0 | 0 | 0 | 0 | 0 | 0 | 0 | 0 | 2 |
| **RBMS1** | 0 | 2 | 0 | 0 | 0 | 0 | 1 | 0 | 0 | 0 | 0 |
| **SFPQ** | 0 | 0 | 0 | 2 | 0 | 1 | 0 | 0 | 0 | 0 | 0 |
| **SRSF9** | 0 | 0 | 0 | 0 | 0 | 0 | 1 | 0 | 0 | 2 | 0 |
| **ANKHD1** | 0 | 0 | 0 | 0 | 0 | 2 | 0 | 0 | 0 | 0 | 0 |
| **CPEB2** | 0 | 0 | 1 | 1 | 0 | 0 | 0 | 0 | 0 | 0 | 0 |
| **FXR1** | 0 | 0 | 0 | 0 | 0 | 0 | 0 | 2 | 0 | 0 | 0 |
| **FXR2** | 0 | 0 | 0 | 0 | 0 | 0 | 2 | 0 | 0 | 0 | 0 |
| **G3BP2** | 0 | 0 | 0 | 0 | 0 | 0 | 0 | 1 | 1 | 0 | 0 |
| **HNRNPCL1** | 0 | 0 | 2 | 0 | 0 | 0 | 0 | 0 | 0 | 0 | 0 |
| **HNRNPU** | 0 | 0 | 0 | 1 | 0 | 0 | 0 | 0 | 1 | 0 | 0 |
| **KHDRBS2** | 0 | 0 | 0 | 0 | 0 | 0 | 0 | 0 | 0 | 0 | 2 |
| **KHDRBS3** | 0 | 0 | 0 | 0 | 0 | 0 | 0 | 0 | 0 | 0 | 2 |
| **MSI1** | 0 | 0 | 0 | 0 | 0 | 0 | 0 | 2 | 0 | 0 | 0 |
| **RBFOX1** | 0 | 0 | 0 | 0 | 0 | 0 | 0 | 2 | 0 | 0 | 0 |
| **RBM41** | 0 | 0 | 0 | 0 | 2 | 0 | 0 | 0 | 0 | 0 | 0 |
| **RBM5** | 2 | 0 | 0 | 0 | 0 | 0 | 0 | 0 | 0 | 0 | 0 |
| **RBM6** | 0 | 2 | 0 | 0 | 0 | 0 | 0 | 0 | 0 | 0 | 0 |
| **RBMS3** | 0 | 0 | 0 | 0 | 0 | 2 | 0 | 0 | 0 | 0 | 0 |
| **CNOT4** | 0 | 0 | 0 | 0 | 0 | 0 | 1 | 0 | 0 | 0 | 0 |
| **HNRNPM** | 0 | 0 | 0 | 0 | 0 | 0 | 0 | 1 | 0 | 0 | 0 |
| **KHDRBS1** | 0 | 0 | 0 | 0 | 0 | 0 | 0 | 0 | 0 | 0 | 1 |
| **PABPN1** | 0 | 0 | 0 | 0 | 0 | 0 | 0 | 0 | 0 | 0 | 1 |
| **SAMD4A** | 0 | 0 | 0 | 0 | 0 | 0 | 0 | 0 | 0 | 1 | 0 |
| **SNRPA** | 0 | 0 | 0 | 0 | 0 | 0 | 0 | 0 | 0 | 1 | 0 |
| **TUT1** | 0 | 1 | 0 | 0 | 0 | 0 | 0 | 0 | 0 | 0 | 0 |
| **ZNF638** | 0 | 0 | 0 | 0 | 0 | 1 | 0 | 0 | 0 | 0 | 0 |

**Supplementary Table S6:** CircRNA-miRNA-mRNA regulatory network for *ci-Ins2* (486 nt)
